# Nonlinear spatial integration allows the retina to detect the sign of defocus in natural scenes

**DOI:** 10.1126/sciadv.adq6320

**Published:** 2025-08-08

**Authors:** Sarah Goethals, Awen Louboutin, Samy Hamlaoui, Tom Quetu, Samuele Virgili, Matias Alejandro Goldin, Konogan Baranton, Olivier Marre

**Affiliations:** ^1^ Groupe Lens Innovation, R&D Life and Light Science, EssilorLuxottica, Paris, France.; ^2^ Institut de la Vision, Sorbonne Université, INSERM, CNRS, Paris, France.; ^3^ Groupe Lens Innovation, Digital Innovation, EssilorLuxottica, Paris, France.

## Abstract

Eye growth is regulated by the visual input. Many studies suggest that the retina can detect whether a visual image is focused in front of or behind the back of the eye and modulate eye growth to bring it back to focus. How can the retina distinguish between these two types of defocus? Here, we simulated how eye optics transforms natural images and recorded how the isolated retina responds to different types of simulated defocus. We found that some ganglion cell types could distinguish between an image focused in front of or behind the retina by estimating spatial contrast. Aberrations in the eye optics made spatial contrast, but not luminance, a reliable cue to distinguish these two types of defocus. Our results suggest a mechanism for how the retina can estimate the sign of defocus and provide an explanation for several results aiming at mitigating strong myopia by slowing down eye growth.

## Teaser

The retina can easily detect whether the eye is too small or too big thanks to the imperfections of the eye optics.

## INTRODUCTION

Our retina is organized in two dimensions and receives a projection of our three-dimensional (3D) visual world, which should presumably make the information about depth ambiguous. However, even with a single eye, there are instances where we can extract information about depth to bring the image reaching the retina into focus.

First, the accommodative reflex aims at keeping the image sharp by changing the focus. The ciliary muscles reshape the crystalline lens to move the focal plane and focus the image on the retina. This reflex can be triggered solely based on monocular cues [reviewed in (*1*)]. Several studies have shown that multiple features of the eye optics may provide cues about the sign of defocus (i.e., whether the focal plane is in front of or behind the retina) and the location of the focal plane, in particular its aberrations (*2*–*5*).

Second, at a longer timescale, the eye needs to constantly adjust its axial length to the optical power of the eye optics to ensure proper image focus on the retina, a process called emmetropization. The visual input is the main regulator of this homeostatic process (*6*). Previous works have shown that eye growth can be perturbed by modifying the visual input (*7*). In young animals, placing a positive lens in front of an emmetropic eye focuses the image in front of the retina (positive defocus) and it slows the growth rate to compensate for the imposed defocus (*8*–*14*). On the other hand, placing a negative lens in front of an emmetropic eye focuses the image behind the retina (negative defocus) and accelerates its growth rate (*8*, *10*, *12*, *13*, *15*–*19*). These studies show that, during development, the eye is able to compensate for an imposed defocus to become emmetropic. Furthermore, a large body of work has shown that excessive eye elongation can also be induced by strongly degrading the visual input [reviewed in (*7*)].

In both cases, the sign of defocus needs to be detected from the image projected to the retina. How this feat is achieved by neural circuits is unclear (*20*). In the case of the accommodative reflex, the stimulus is first processed in the retina, whose ganglion cells send information to the brain. Eventually, the Edinger-Westphal nucleus controls the ciliary muscles. As a first step in visual processing, the retina may help in determining the sign of defocus to drive the accommodative reflex (*21*), but its exact role is unclear.

The retina plays a role in emmetropization that probably goes beyond a preprocessing step. Several studies strongly suggest that the retina can modulate eye growth and that feedback from the brain is not necessary. Sectioning the optic nerve in chicks does not prevent the acceleration of growth rate in response to an imposed positive defocus (*22*) nor does it prevent excessive eye growth in response to visual input degradation (*23*–*26*). Moreover, blocking action potentials from retinal ganglion cells (RGCs) by intravitreal injection of tetrodotoxin (TTX) does not prevent eye elongation in chicks (*27*) and tree shrews (*28*) that underwent visual input degradation. Last, depriving only half of the visual field results in local eye elongation in the corresponding half of the eyeball (*29*, *30*), and eye growth seems to be mostly driven by the peripheral retina (*31*–*33*). Together, these results suggest that eye elongation is driven by retinal mechanisms that compute the sign of defocus and that operate locally (*20*), along with other mechanisms that might also modulate eye growth [light spectrum and intensity (*7*, *34*)], melanopsin signaling and intrinsically sensitive RGC’s activity (*35*, *36*), photoreceptors [reviewed in (*37*)] and glial mechanisms [reviewed in (*38*)].

Retinal computations may thus play a key role to detect the sign of defocus and distinguish an image focused in front of the retina from an image focused behind it. This requires the retina to extract features that are informative about the sign of defocus. How it can achieve this is unclear.

Most previous studies addressed this issue with in vivo experiments where the respective contribution of the eye optics and of the retina could not be easily separated. Here, we developed a hybrid approach combining optics, electrophysiology, and modeling to investigate how the eye optics influences retinal cell’s activity. We simulated the optics of the mouse eye with a detailed optical model for different positions of the focal point with respect to the retina. We then transformed natural images with this optics (*39*) to obtain realistic defocused retinal images. We dissected the mouse retina to remove the real optics of the eye, projected the defocused images on the ex vivo retina, and recorded the impact of the sign of defocus on the RGC’s activity. We identified two types of RGCs whose activity systematically decreases with a more positive defocus, independently of the specific visual input. This shows that some RGCs can robustly detect the difference between an image focused in front and behind the retina. By modeling the responses of these cells, we found that they compute local spatial contrast (LSC) (*40*) inside their receptive field. We show that computing the LSC is sufficient to detect the sign of defocus because of spherical aberrations of the eye optics that make it very different for positive and negative defocus. Our work suggests a strategy for the retina to determine the sign of defocus and provides an explanatory basis for several results in the literature concerning myopia mitigation strategies that aim at slowing down the eye growth.

## RESULTS

### Simulating the mouse eye optics

To measure the impact of the eye optics on retinal activity, we designed a hybrid approach: We simulated in detail how natural images are transformed by the optics of the eye. We then projected these transformed images on the retina in vitro and measured how RGC responses were influenced by the optical transformation. Some studies suggest that emmetropization is preserved during optic nerve section (*25*), suggesting that RGCs do not play a role in this process. However, RGCs read out the activity of most of the retinal network. By asking whether they carry information about the sign of defocus, we expect to determine the computations that are biologically plausible for the retinal circuit and that can give information about the sign of defocus.

The experiments were done in retinas of mice, an animal model in which eye growth can be perturbed (*13*, *14*), ganglion cell activity can be recorded (*41*), and the eye optics has been characterized (*42*, *43*).

**Fig. 1.**
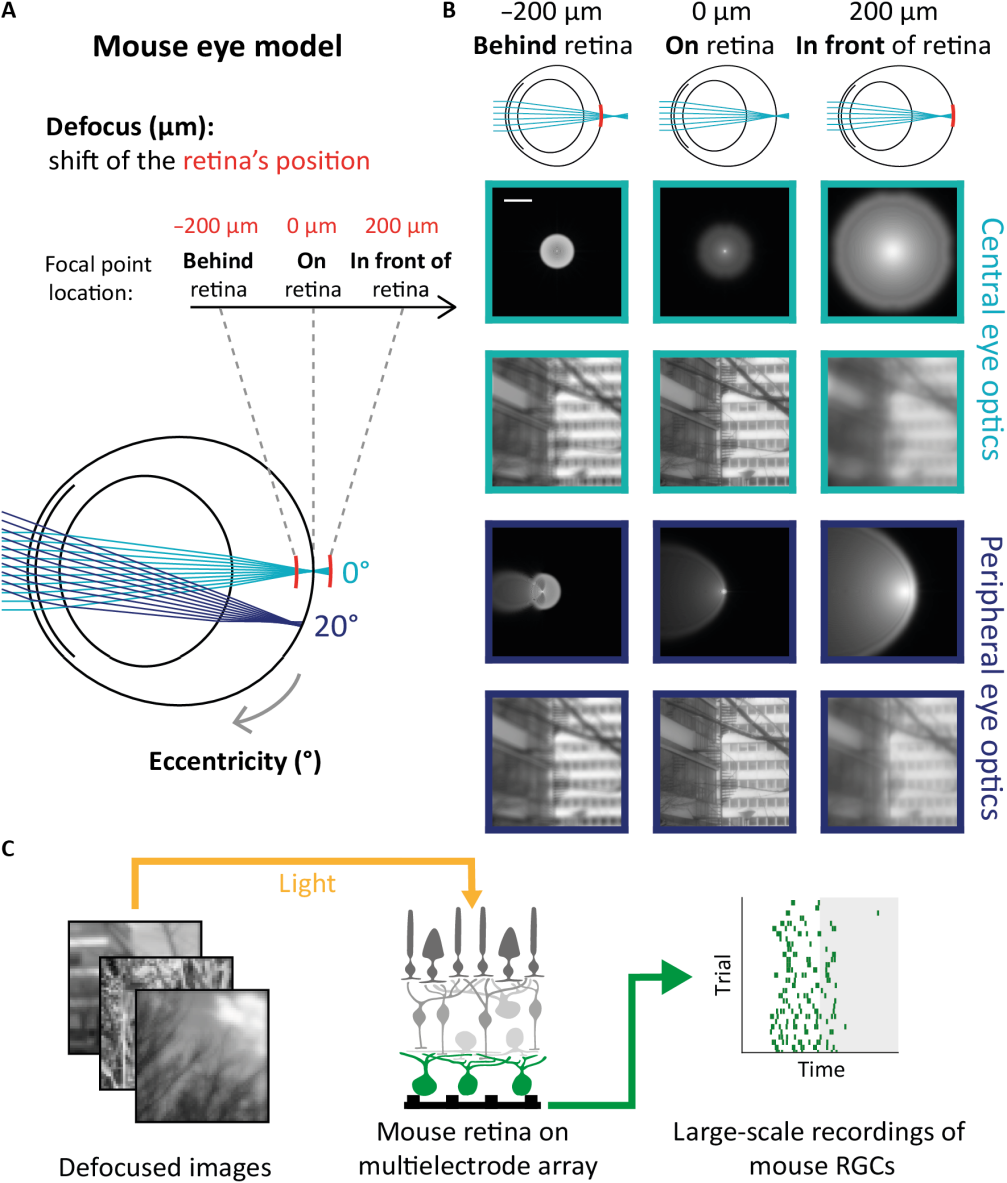
Mouse eye model to simulate retinal images. (**A**) Mouse eye model and its parameters. First, the light source eccentricity: Light rays can reach the retina in the center (0°, light blue) or periphery (20°, dark blue). Second, the position of the retina with respect to the focal point (defocus, in μm). In the model, we kept the eye optics fixed and simulated a change of defocus by moving the position of the retina. Negative values of defocus correspond to the retina being shifted in front of the focal point, and therefore the image becomes focused behind the retina (hyperopic defocus). Positive values of defocus correspond to the retina being shifted behind the focal point, and therefore the image becomes focused in front of the retina (myopic defocus). (**B**) Top: Mouse eye model when the image is focused behind (left), on (center), or in front of (right) the retina. Bottom: PSFs (first and third rows) and the corresponding retinal image (second and fourth rows) for simulated central (0°, light blue) and peripheral (20°, dark blue) eye optics, when the image is focused behind (left), on (center), or in front of (right) the retina. (**C**) Experimental preparation. Natural images transformed by the eye optics (left) are projected on an ex vivo mouse retinal flattened on a MEA (center) to record RGC’s responses (right). RGCs are represented in green. The raster plot on the right is the spiking activity of one cell in response to 30 presentations of the same image during 300 ms. The gray area corresponds to the presentation of a gray frame during 300 ms. Credit for the natural images shown here goes to H. Van Hateren (*87*).

Our detailed model of the mouse eye optics (see Materials and Methods) takes into account optical aberrations. As a result, and in contrast with a model with no aberrations, the image projected on the retina can be different if the image is focused in front of the retina or behind. To illustrate this, we simulated how a point source of light transformed by the eye optics appears on the retina, which is usually called the point spread function (PSF) of the optical system. It represents a local linear approximation of the transformation imposed on the stimulus by the eye optics. This PSF will depend on the position of the focal point with respect to the retina and on the eccentricity (in degrees of visual angle) of the spot of light. Note that, in our model, the eye optics is fixed and the defocus is simulated by moving the position of the retina with respect to the focal point (Fig. 1A). For various eccentricities, the PSF of an image focused in front of the retina was always different from the PSF corresponding to an image focused behind the retina (Fig. 1B). The PSF spread around the main focal point is much broader for a focus in front of the retina. For simulated peripheral eye optics, the PSF spreads around the main focal point mainly in one direction along the horizontal axis (Fig. 1B). When convolving these PSFs with natural images, the change of focus had a visible impact on the resulting image falling on the photoreceptor plane: The contrast and the amount of blur were different if the focus was positive (focal plane in front of the retina) or negative (focal plane behind the retina).

The images transformed by the eye optics will thus be different if the image is focused in front of or behind the retina. Note however that the PSFs were shown in log scale, which amplifies these differences. It is thus unclear if the retina can detect this difference, in particular when stimulated with natural images.

### Some types of mice RGCs detect the sign of defocus

**Fig. 2.**
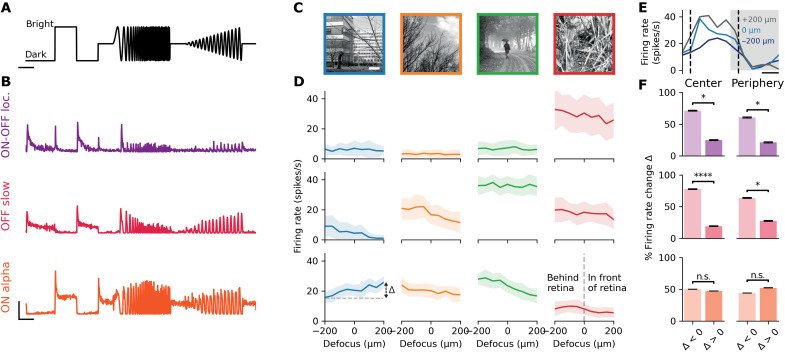
Specific types of retina ganglion cells could signal the sign of defocus. (**A**) Chirp stimulus (see Materials and Methods). Scale bar, 2 s. (**B**) Example RGC response to the chirp stimulus. Top: ON-OFF local; middle: OFF slow; bottom: ON alpha. Horizontal scale bar, 2 s. Vertical scale bar, 20 spikes/s. (**C**) Four images that were defocused and flashed on the retina during MEA experiments. Scale bar, 500 μm. (**D**) Firing rate as a function of defocus for the images in (C) and for three example cells, for simulated peripheral eye optics (20°). Top: ON-OFF local; middle: OFF slow; bottom, ON alpha. Data are represented as means ± SD. (**E**) Raw response of an ON alpha cell to the “blue” image for simulated peripheral eye optics (20°): firing rate during image (white area) and gray frame (gray area) presentation for a defocus of −200 μm (dark blue), 0 μm (blue), and 200 μm (light blue). The vertical dashed lines represent the time range during which we measure the firing rate. (**F**) Proportion of images and cells leading to a firing rate change ∆ [between defocus of +200 μm and defocus of −200 μm; see (D), bottom left], that is strictly negative (left bar plot) or strictly positive (right bar plot). Left (right) column: simulated central (peripheral) eye optics (0°) (20°). Top: ON-OFF local (*N* = 7 cells x 4 images), center: *P* = 1 × 10^−2^; periphery: *P* = 1 × 10^−2^. Middle: OFF slow (*N* = 9 cells x 4 images), center: *P* = 5 × 10^−5^; periphery: *P* = 1 × 10^−2^. Bottom: ON alpha (*N* = 55 cells x 4 images), center: *P* = 0.1; periphery: *P* = 0.9). One-sided Wilcoxon signed-rank test. Credit for the natural images shown here goes to (*87*). *P ≤ 0.5; ****P ≤ 0.0001; n.s., not significant.

To answer this question, we projected these defocused images on mice retinas ex vivo and recorded RGC’s electrical responses to different optical transformations with a multielectrode array (MEA) (Fig. 1C; see Materials and Methods). We then measured how RGC’s firing rate varies with the simulated defocus, simulating different eccentricities to test the robustness of our results. Because the peripheral retina has been identified as the main driver of eye growth (*31*–*33*), we decided to focus our analyses on the effects of peripheral eye optics. For the sake of robustness, we additionally show the main results for central eye optics. In addition, we presented a chirp stimulus (Fig. 2A) to classify RGCs depending on their response to full-field bright and dark stimulus (see Materials and Methods) (Fig. 2B).

We found that, for many cells, the change in the simulated defocus was often enough to evoke a change in their firing rate (Fig. 2, C to E). The difference in firing rate was visible when switching from a positive defocus (focus in front of the retina) to a negative defocus (focus behind the retina) (Fig. 2E). The changes in image properties induced by the change in PSF are thus sufficient to evoke a change in the RGC response.

Presumably, the sign of this firing rate difference should depend on the content of the image inside the receptive field of the cells, and this is what we found for many of them. However, unexpectedly, we found two RGC types [OFF slow and ON-OFF local (*44*)] (Fig. 2B) where the firing rate almost never increased when the defocus changed from negative to positive: It always decreased or stayed constant, for the images we tested (Fig. 2, C and D), across all simulated eccentricities of the optical model, and across all the cells of the same type (Fig. 2F) [ON-OFF local, *N* = 7 cells x 4 images, center: 71 negative firing rate change ∆ (decreasing firing rate) versus 25% positive firing rate change ∆ (increasing firing rate), *P* = 1 × 10^−2^, periphery: 61 versus 21%, *P* = 1 × 10^−2^; OFF slow, *N* = 9 cells x 4 images, center: 78% versus 19%, *P* = 5 × 10^−5^, periphery: 64 versus 28%, *P* = 1 × 10^−2^, one-sided Wilcoxon signed-rank test]. This suggests that these cells could reliably detect the sign of defocus.

Note that we found this behavior in RGCs from other types (see Discussion), but this was not the case for all the cell types. ON alpha cells, for example, did not show this property; their firing rate could either increase or decrease when the sign of defocus changed, depending on the natural image displayed (Fig. 2F, ON alpha: *N* = 55 cells x 4 images). Therefore, the systematic decrease property is specific to some cell types and not shared by all RGCs.

**Fig. 3.**
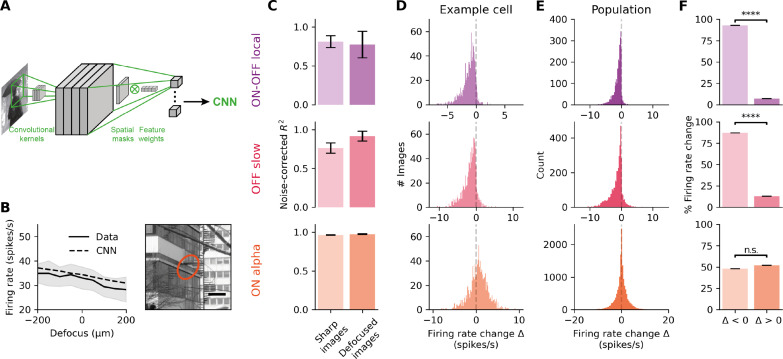
A CNN model accurately predicts RGC response to defocused images and confirms that two types almost always decrease their activity when the focus switched from negative to positive. Data of (B), (D), (E), and (F) are shown for simulated peripheral eye optics (20°). For (C), all eccentricities and defocusses were pooled. (**A**) Schematic of the CNN model architecture. Adapted from (*45*). (**B**) Example of an ON alpha cell’s response (full lines) to the images shown on the right, and the predictions of the CNN model (dashed lines). The orange ellipse on the images is the receptive field of the cell. Scale bar, 200 μm. (**C**) Average performance of the model at predicting the response to repeated not defocused (left) and repeated defocused natural images (right). Data from *N* = 3 ON-OFF local cells (top), *N* = 4 OFF slow cells (middle) and *N* = 15 ON alpha cells (bottom). Data are represented as means ± SEM. (**D**) Distribution over *N* = 1000 images of the change in predicted firing rate ∆ between defocus of 200 and −200 μm for an ON-OFF local (top), an OFF slow (middle), and an ON alpha (bottom) example cells. (**E**) Same as (D) for all the modeled cells. Top: ON-OFF local (*N* = 3 cells x 1000 images). Middle: OFF slow (*N* = 4 cells x 1000 images). Bottom: ON alpha (*N* = 15 cells x 1000 images). (**F**) Proportion of images and cells leading to a firing rate change ∆ (between defocus of +200 μm and defocus of −200 μm), that is strictly negative (left bar plot) or strictly positive (right bar plot). *N* is as for (D). Top: ON-OFF local (*P* = 0); middle: OFF slow (*P* = 0); bottom: ON alpha (*P* = 0.1); one-sided Wilcoxon signed-rank test. ****P ≤ 0.0001; n.s., not significant.

We have found that, for these two types of RGCs, firing systematically decreases with defocus. To test whether this decrease is systematic over a very large set of images, we built a model to predict how these RGCs may respond to other defocused natural images. We used a convolutional neural network (CNN) model of the retina that has been previously shown to predict well ganglion cell responses to natural images (*45*) (Fig. 3A). The model was trained on the RGC’s responses to a large set of 3010 not defocused natural images (see Materials and Methods). Its performance was evaluated on a set of 30 held-out repeated not defocused natural images (“test set”). For both types, this CNN model accurately predicts RGC’s response to not defocused natural images (ON-OFF local: *R*^2^ = 0.81 ± 0.13; OFF slow: *R*^2^ = 0.76 ± 0.13; ON alpha: *R*^2^ = 0.96 ± 0.02) (Fig. 3C). It could also generalize and predict with comparable performance RGC’s response to defocused images (ON-OFF local: *R*^2^ = 0.77 ± 0.3; OFF slow: *R*^2^ = 0.92 ± 0.06; ON alpha: *R*^2^ = 0.97 ± 0.02) (Fig. 3C). Some cells were discarded from this analysis because RGCs need to have very stable activity all along the presentation of the natural images stimulus to be properly modeled by the CNN (see Materials and Methods).

We then used this model to test whether the cell’s firing rate varies in the same direction whatever the specific image. We predicted RGC’s responses to thousand defocused natural images and examined the distribution of the change in firing rate between a positive defocus and a negative defocus (∆) (Fig. 3, D and E). For the ON-OFF local type, 92.7% of the images across the cell population lead to a negative change in firing rate, and this proportion is 87.1% for OFF slow cells (Fig. 3F). It shows that that these cell’s firing rate is strictly lower for a positive defocus than for a negative defocus, for almost all natural images. On the contrary, ON alpha cell’s firing rate did not show this effect: When the defocus changed from positive to negative, firing rate could either decrease or increase depending on the natural image, with equal probabilities (48.0% versus 51.2%) (Fig. 3F; see also the almost symmetrical distribution of the change in firing rate in Fig. 3E).

To summarize, the CNN model predictions confirm that some types of RGCs almost always decrease their firing rate with defocus, whatever the specific visual input, and that this is a specific property of some type but not ubiquitous among ganglion cells. Our results suggest that these cell types can be used to robustly detect the sign of defocus during natural image stimulation. From now on, we will call them “defocus detectors.”

### Defocus detector cells encode the LSC

We next wondered how defocus detectors can actually perform the detection of the sign of defocus. Presumably, they have to extract a specific feature from the natural image, which will unambiguously tell the sign of defocus. To answer this question, we extracted the values of the mean intensity (MI) and contrast inside the receptive fields (most studied low level image statistics) of the two cell types identified above, across different images and eye model parameters and used them as inputs to simple models to study whether they could reproduce our previous results.

**Fig. 4.**
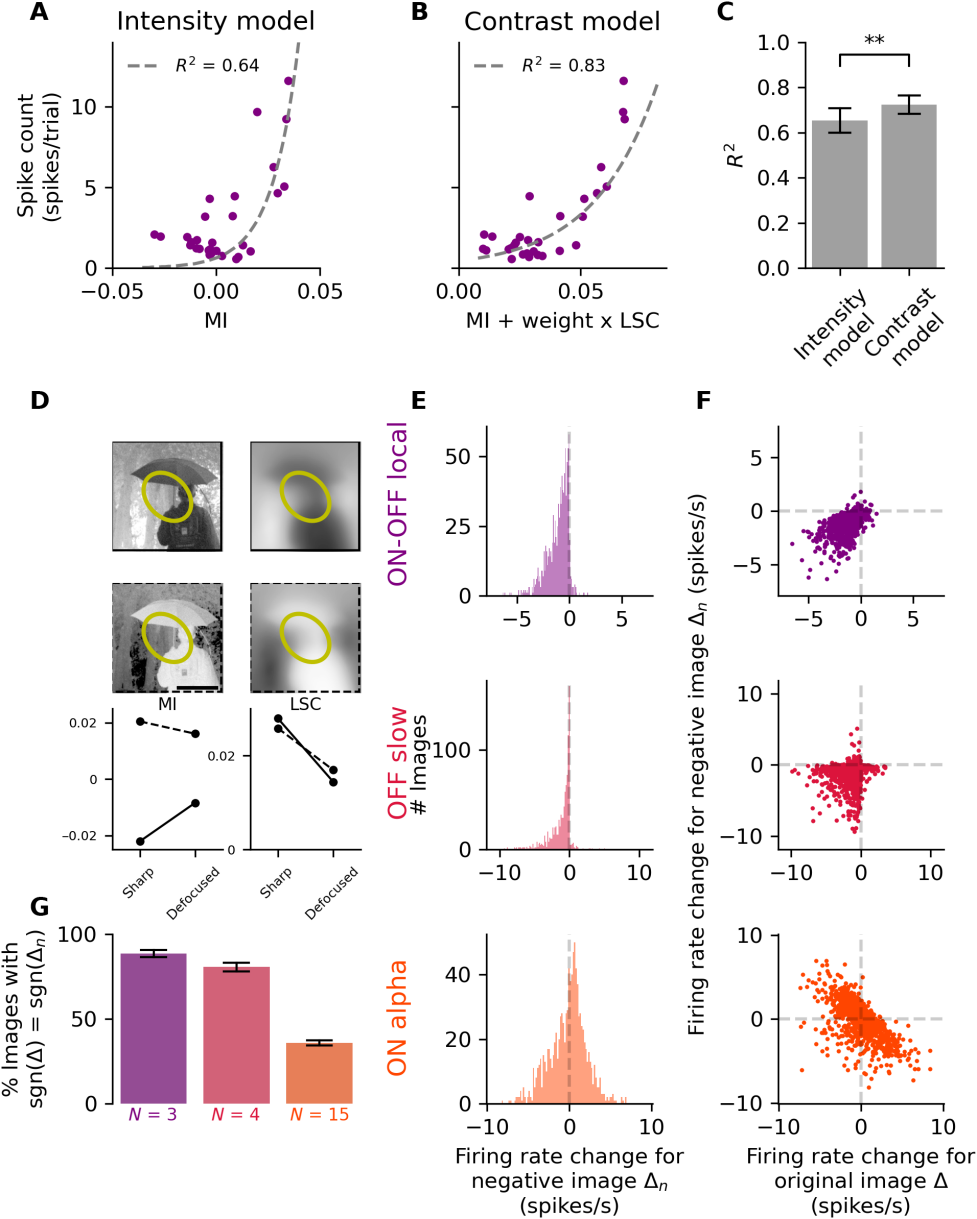
A simple contrast model and the predicted responses to negative images confirm that defocus detectors encode the LSC. Data of (D), (E), (F), and (G) are for simulated central eye optics (0°). For simulated peripheral eye optics, see fig. S2. (**A**) Average firing rate as a function of MI in the receptive field for an ON-OFF local RGC for the test set images (see Materials and Methods). The gray line represents the fit of the intensity model. (**B**) Same as (A) for the MI + LSC. (**C**) Distributions of the coefficient of determination *R*^2^ for the intensity model (left, *R*^2^ = 0.65 ± 0.05) and the spatial contrast model (right, *R*^2^ = 0.72 ± 0.04) over all defocus detector (*N* = 15, *P* = 3 × 10^−3^, one-sided Wilcoxon signed-rank test). Data are represented as means ± SEM. (**D**) Sharp (left) and defocused (right, defocus = 200 μm) image (top) and its bright-dark inverse (middle), with the receptive field of an example cell. Scale bar, 200 μm. Bottom: MI and LSC for the original (full line) and the negative (dashed line) image. (**E**) Distribution over 1000 images of the change of firing rate ∆ between 200 and −200 μm in response to the negative defocused images. Top: ON-OFF local cell. Middle: OFF slow cell. Bottom: ON alpha cell. (**F**) Change of firing rate in response to the negative images versus change of firing rate in response to the original images for the cells of (E). Each dot represents an image. (**G**) Average (over cells, means ± SEM) of the proportion of images leading to a firing rate change ∆*_n_*, that as the same sign as the firing rate change ∆ for the original image. Left: ON-OFF local; middle: OFF slow; right: ON alpha. **P ≤ 0.01.

We first learned a classical linear nonlinear model (*40*), which takes as an input the MI inside the receptive field center and aims at predicting the response (“Intensity model,” Fig. 4A). The parameters of this model were learned from the response of each cell to a set of flashed not defocused natural images (same set as for the CNN; see Materials and Methods). Following Liu and colleagues (*40*), we then tested whether including second-order statistics as an input to the model could improve the performance (Fig. 4B). For each image presented, we measured the LSC (see Materials and Methods), which is the SD of the pixel intensity in the receptive field center. When this LSC was added as an input to the intensity model described above, it improved the prediction performance (Fig. 4C), suggesting that these cells (at least partially) encode the LSC.

In a previous work, we have shown that some ganglion cells encode LSC or a mixture of MI and LSC (*45*). To further confirm our results, we analyzed the defocus detectors with the same tools as in this study and found further evidence that they behaved like LSC encoders or encoded a mix of MI and LSC (fig. S1).

The MI and the LSC have different behaviors when turning an image to its negative: Although this transformation will substantially change the MI, it will leave the LSC constant because LSC is the SD of the intensity inside the receptive field (Fig. 4D). Moreover, if we apply a strong blur to an image, it will make it closer to gray (Fig. 4D). If the MI inside a receptive field was originally brighter than gray, it will decrease. If we take the negative of this image patch, blurring it the same way will increase its intensity, because it will also drive the MI toward gray. However, LSC will decrease when blurring this image, for both the positive and negative images. Local intensity and LSC have thus very different behaviors in response to this strong blur. Therefore, we reasoned that, if these defocus detectors are mostly explained by MI, turning every image to its negative should substantially change their way to respond to defocus. Conversely, if they are mostly driven by changes in LSC, this transformation should not affect qualitatively the way they respond to defocus.

We tested how defocus detector cells behaved when turning all the images in their negatives. We found that this did not alter the way they responded to defocus. For negative images, they still decreased their firing rate when responding to defocus (Fig. 4E). The firing rate changed in the same direction when changing the defocus for a given image and its negative (Fig. 4, F and G, same direction: ON-OFF local: 88.6 ± 1.7%, OFF slow: 80.6 ± 2.3%). On the contrary, firing rate of ON alpha cells could equally increase or decrease when defocus changed from negative to positive (Fig. 4E), as observed above (Fig. 3D). For most images, the original and the negative versions will trigger changes of firing rate in opposite directions with the defocus (same direction: 35.9 ± 1.5%) (Fig. 4, F and G), which is indicative of cells whose activity is driven mostly by local MI (*45*).

Overall, these results suggest that the cells could encode the sign of defocus because their responses were driven by changes in the LSC. Note that some of these cells were not only modulated by LSC but rather by a mix between MI and LSC (fig. S1). Moreover, an alternative explanation to these results could be that ON-OFF cells respond both to light increase and light decrease with respect to the average gray. In this case, these cells would not encode LSC, but their activity would be correlated with LSC and better explained by LSC than MI. In any case, our analysis suggests that LSC, or something correlated to LSC, was helpful to make their response to the sign of defocus more independent of the content of the image.

### LSC allows to detect the sign of defocus

**Fig. 5.**
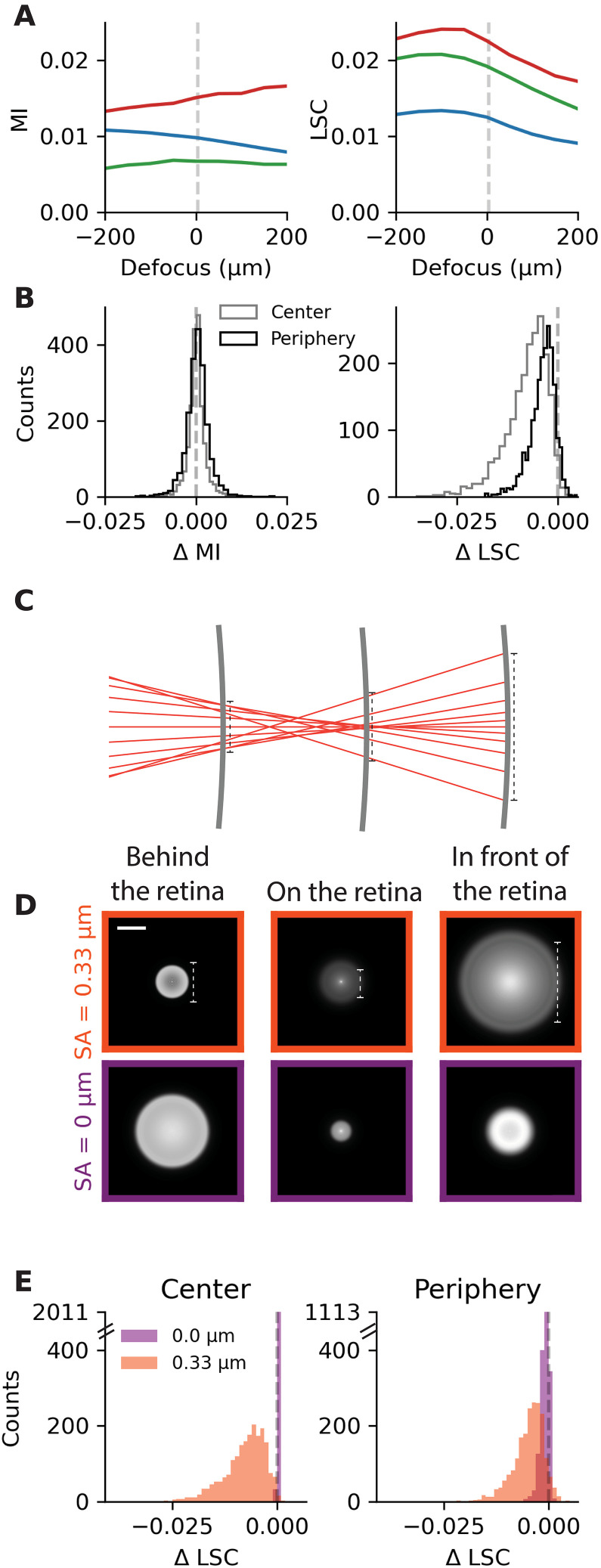
Spherical aberrations are necessary to detect the sign of defocus with the computation of LSC. (**A**) MI (left) and LSC (right) as a function of defocus for three different images (red, green, and blue curves, same color code as in Fig. 2C) and simulated peripheral eye optics (20°), computed in the receptive field of an example cell. (**B**) Distribution of the difference in MI (left) and the difference in LSC (right) over *N* = 511 cells x 4 images, between defocus of −200 and 100 μm. Gray (black), in images corresponding to the simulated central (peripheral) eye optics. (**C**) Schematic illustrating the spherical aberrations in a mouse eye model with pupil diameter = 1.4 mm. The middle gray thick line represents a retina located at the focal point. The left (right) gray line represents a situation where the focal point would be located behind (in front of) the retina. (**D**) Top row: PSFs with spherical aberrations (SA = 0.33 μm, PSF shown in log scale) corresponding to the positions of the three different retinas in (C), for simulated central eye optics (0°). Bottom row: PSFs at the same positions but without spherical aberrations. Scale bar, 50 μm. (**E**) Distribution of the difference in LSC over *N* = 511 cells x 4 images, with spherical aberrations (orange) and without (purple). Left (right): In images corresponding to the simulated central (peripheral) eye optics.

Because the LSC seems to be necessary to understand the responses of the two cell types we characterized as defocus detectors, we tested whether computing this LSC is sufficient to estimate the sign of defocus. Starting with an example cell, we compare how the MI and the LSC vary with defocus (Fig. 5A). Depending on the image, the MI can increase (red and green lines) or decrease (blue line) with defocus. It suggests that the average intensity inside the receptive field is not a reliable clue to detect the sign of defocus. On the contrary, for the three example images shown in Fig. 5A, the LSC reaches a maximum for mild negative defocus and decreases with more positive defocus. It suggests that the LSC is a better clue to detect the sign of defocus, as its variation with defocus does not show dependence on the specific image.

To confirm this observation at the population level, we measured the MI and the LSC in defocused images in the receptive fields of many cells across six experiments (*N* = 511). To quantify the change of MI and LSC across the cell population, we compute the difference in MI (∆MI) and LSC (∆LSC) between 200 and −100 μm, around the maximum of LSC observed in Fig. 5A. We find that ∆MI can be positive or negative, depending on the cell, meaning that the MI can increase or decrease with defocus (Fig. 5B). It confirms that the mean intensity is not a reliable clue to detect the sign of defocus. On the contrary, ∆LSC is negative for almost all cells and images, meaning that the LSC almost always decreases with more positive defocus (Fig. 5B). It confirms that the LSC is a reliable clue to detect the sign of defocus in natural images transformed by the eye optics and shows that computing the LSC allows to detect the sign of defocus.

Why is the LSC so different when the image is focused in front of (positive defocus) or behind the retina (negative defocus)? We have shown above that the PSFs are very different in front of and behind the retina (Fig. 5D). These PSFs differ because the optics of the eye is not perfect. Of most importance are the spherical aberrations: All the rays that go through a lens do not focus at one single focal point (Fig. 5C). Marginal rays (near the pupil boundary) focus ahead of paraxial rays (close to the center axis), which leads to positive spherical aberrations. If the marginal rays are focused behind the paraxial rays, this will cause negative spherical aberrations. This makes the blur asymmetrical with respect to the position of the retina. When the retina is in front of the focal point (left part of the schematic C), rays accumulate at the edge of the PSF, creating an intense ring at the border of the PSF (Fig. 5D, left). When the retina is located behind the focal point (right part of the schematic), rays at the edge of the PSFs spread on a larger area, so that the peak of the PSF is more intense that the edge, and the PSF is spread in a larger area (Fig. 5D, right). This difference allows a simple computation like the LSC to be sufficient to detect the sign of defocus. To test directly whether spherical aberrations are important for defocus detection, we made the spherical aberration variable in the simulated mouse eye model by changing the shape of the anterior cornea, which is the largest contributor to spherical aberrations. This means that its radius of curvature is not constant anymore but varies as the distance from the center increases. Totally removing the spherical aberration in the model made the PSF in front of or behind the focal point much more similar (Fig. 5D). As a consequence, the LSC stopped decreasing systematically when switching from negative to positive defocus, and it became impossible to detect the sign of defocus by computing the LSC (Fig. 5E; see also fig. S3).

The spherical aberrations in the eye optics thus provide robust cues for defocus detection, as already proposed by Thibos and colleagues (*46*). To detect the sign of defocus, a realistic strategy for the retina could be to rely on spherical aberrations and on the computation of LSC.

### LSC in human retinal images

**Fig. 6.**
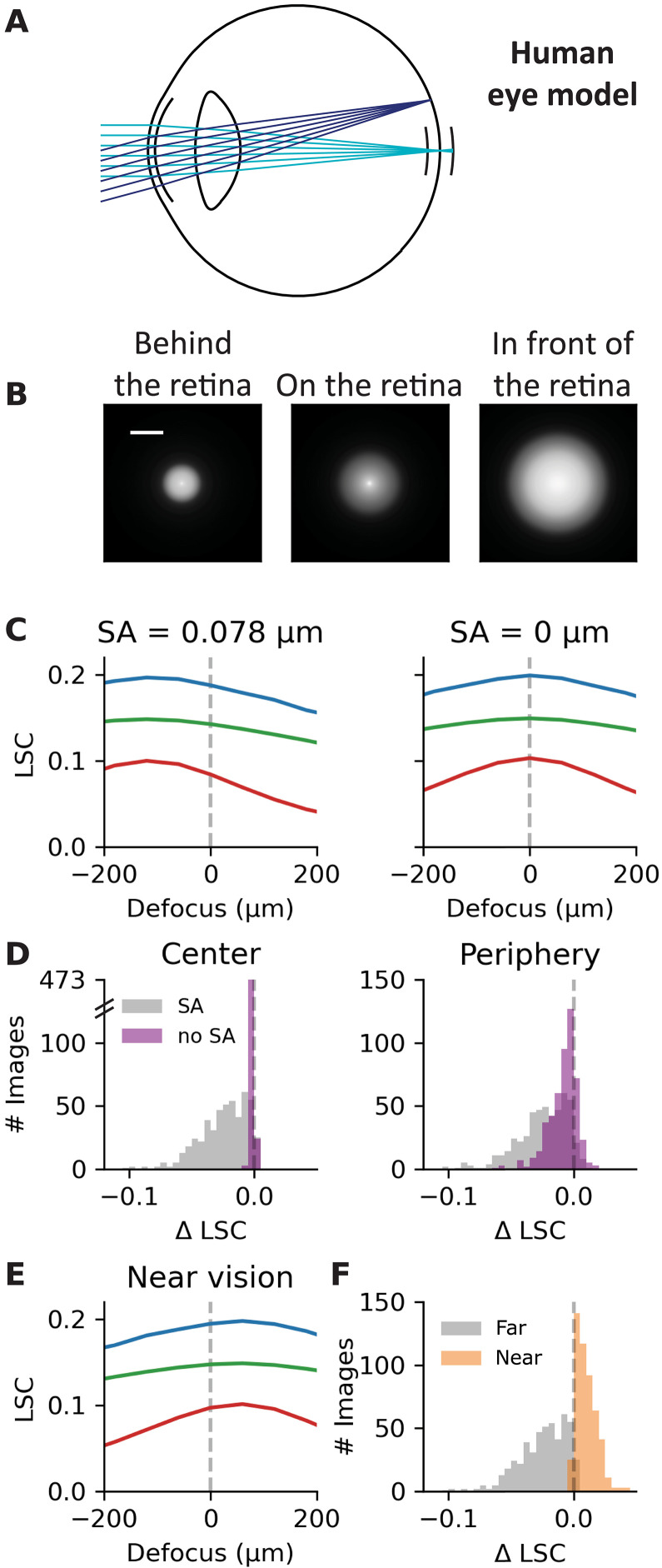
Reduced spherical aberrations make the LSC more ambiguous to determine the sign of defocus. In (C), (E), and (F), the LSC is calculated in images corresponding to simulated central eye optics (0°). (**A**) Human eye model. The light rays can reach the retina in the center (0°, light blue) or periphery (20°, dark blue) of the retina. The pupil diameter is 5 mm. (**B**) PSFs of the simulated central eye optics when the image is focused behind (left), on (center), or in front of (right) the retina. Scale bar, 50 μm. (**C**) LSC as a function of defocus for three example images (color code as Fig. 2C) with (left) and without (right) spherical aberrations. (**D**) Distribution of the difference in LSC between 200 and −200 μm over *N* = 500 images, with spherical aberrations (gray, SA = 0.078 μm) and without (purple). Left (right): In images corresponding to the simulated central (peripheral) eye optics. (**E**) LSC as a function of defocus for three example images (color code as Fig. 2C) in near vision (focus proximity = 2.5 D). (**F**) Distribution of the difference in LSC between 200 and −200 μm over *N* = 500 images, for a model with spherical aberrations and in far vision [gray, same as the gray histogram of (D), left] and a model with spherical aberrations in near vision (orange, focus proximity = 2.5 D, SA = 0.030 μm).

Is the decrease in LSC when the focal point moves in front of the retina a general feature in realistic eye optics or is it specific to the mouse? To tackle this question, we developed a model of the adult human eye (see Materials and Methods) (Fig. 6A). The PSFs of the simulated central eye optics show the same features as in the mouse eye model: The PSF corresponding to the focal point being behind the retina is less spread than when the image is focused in front of the retina (Fig. 6B). Consequently, when the image is focused in front of the retina (Fig. 6B, right), the LSC is smaller than when the image is focused behind the retina (Fig. 6B, left) for most of the tested natural images (Fig. 6, C and D) [see also (*46*)]. It suggests that computing the LSC could be a realistic strategy to detect the sign of defocus for the human retina too.

We next tested whether the absence of spherical aberrations was also sufficient to cancel the change of LSC with defocus. If we remove spherical aberrations in the human eye model, the change in LSC is strongly reduced for simulated central eye optics (Fig. 6, C and D). This reduction is less important for peripheral eye optics because off-axis aberrations that are negligible in central vision become substantial in peripheral vision. Therefore, for both central and peripheral eye optics, determining the sign of defocus with the LSC will remain more difficult without spherical aberrations because the change of LSC is reduced (with SA versus without SA, *P* < 0.01 for central and peripheral eye optics, two-sample Kolmogorov-Smirnov test). Overall, these results suggest that the same strategy of computing LSC could work in the human retina too and that spherical aberrations are helpful for this.

In near vision, spherical aberrations are reduced and can even change sign (*46*, *47*). Therefore, we examined the effect of accommodation on the change in LSC with defocus. Simulating near vision in our human eye model, we found that the spherical aberration becomes slightly negative (−0.030 μm at 2.5 D of focus proximity versus 0.078 μm at 0 D with a 5-mm pupil) and that the LSC is higher when the image is focused in front of the retina, for central eye optics (Fig. 6, E and F). For peripheral eye optics (fig. S6), the effect of near vision on the change of LSC is less marked but still significant (far versus near, *P* < 0.01 for central and peripheral eye optics, two-sample Kolmogorov-Smirnov test). If the retina relies on the LSC to determine the sign of defocus, near vision will make this task more difficult.

## DISCUSSION

### Detection of the sign of defocus by retinal cells

We designed a hybrid approach to record mouse RGCs response to realistic defocused images with MEAs. We simulated the optical transformation of the visual input using a detailed eye model that transformed the natural images that we presented to the retinas [see also (*39*)]. We found that spherical aberrations of the eye optics caused an asymmetry between the image focused in front and the image focused behind the retina. Because of these aberrations, computing the LSC allows to detect the sign of defocus. We found ganglion cells that systematically had a stronger response to a focus behind (negative defocus) compared to a focus in front (positive defocus) and that compute the LSC. Estimating MI alone was not sufficient to unambiguously estimate the sign of defocus. It is thus possible to estimate where the focal plane is by relying on two key features, spherical aberrations of the eye optics and LSC computation.

Taking into account the details of the transformation that the eye optics imposes on natural stimuli was necessary to understand how the retina could detect the sign of defocus. Previous works (*48*, *49*) where the defocus was simulated using an artificial lens did not find a difference in ganglion cell’s responses, or are inconclusive, probably because they did not have substantial spherical aberrations. As a result, the responses to negative and positive defocus could not be distinguishable (*48*). The detection of the sign of defocus relies on the specificity of the eye optics. Other works have taken into account these specificities to develop a theoretical method for estimating the defocus in natural images but without the support of neurophysiological data (*50*).

We focused on two ganglion cell types for which every cell’s response decreases when the focus went from negative to positive. We also found ganglion cells from other types that seem able to detect the sign of defocus by computing the LSC (fig. S3), in particular ON-OFF ganglion cells. Note that there might therefore be equivalent ON-OFF bistratified ganglion cells in primates able to perform the same computation. A previous study in mice has shown that a specific ganglion cell type with a long latency seems to be suited for signaling whether an image is in focus or not (*21*), although it was thought that it could not make a difference between positive and negative defocus (*51*). In our study, each natural image was flashed for 300 ms, and this was too fast compared to the latency of this cell type. For this reason, we could not determine in our experiments whether this cell type was also able to encode the sign of defocus. However, using stimuli having different temporal dynamics, and taking into account the spherical aberrations of the eye optics when presenting stimuli like we did in our study, it is possible that this type could also signal the sign of defocus.

The simplicity of the computation of LSC makes it probably widespread across cell types [see also (*45*)]. Our study shows that LSC is a feature that is informative about the sign of defocus and that it can be computed by the retinal circuit. This does not tell which cell types could be causally involved in regulating eye growth, but it puts constraints on possible candidates: Presumably, they should be sensitive to LSC. Although we have studied this LSC computation in ganglion cells, LSC can also probably be computed by some amacrine cell types, especially the ones that receive both ON and OFF inputs [dopaminergic amacrine cells (*52*), vasoactive intestinal polypeptide-expressing amacrine cells (*53*, *54*), nitric oxide synthase–containing amacrine cells (*55*), but also others]. The modulation of eye growth by the retina seems to involve several mechanisms but does not seem to be caused by spiking ganglion cells (*27*, *28*); however, see (*56*). If ganglion cells are not causally involved, one of the retinal cell types that could be causally involved in modulating eye growth to achieve emmetropization would be amacrine cells that are able to compute LSC. Future works will be needed to determine which amacrine cell types can perform this computation.

### A possible strategy to detect the sign of defocus for emmetropization

Our results suggest a possible strategy for the retina to detect the sign of defocus for emmetropization by relying on spherical aberrations and computing LSC: An overall decrease in LSC suggests that the focal plane is in front of the retina, whereas an increase suggests that the focal plane is behind it. The LSC is sufficient for this because the mean LSC changes monotonically around the focal point (Figs. 5 and 6, C and D, and fig. S4A). A practical implementation of a homeostatic regulation of eye growth based on LSC requires to compare the LSC with a reference LSC value at the focal plane: When the measured LSC is lower than the reference, eye growth will be slowed down. The retina could thus compute the mean LSC over a short period of time and over a number of cells and try to reach the reference LSC value by modulating the eye growth. We estimated that the error done if such a strategy was implemented to be below 0.08 D (fig. S4, B and C). Estimating the LSC and using it to modulate eye growth to reach the focal plane is therefore a possible strategy for emmetropization. If the cells that modulate eye growth are also influenced by other features (e.g., the MI in our analyses), a reference cell population responding only to MI might be needed to differentiate effects of defocus and of other stimulus aspects. RGCs responding only to MI have been identified in our work and by others (*45*, *57*). Causal interventions on specific cell types will be necessary to understand better how the LSC is computed to determine the sign of defocus.

Chromatic aberrations could also provide a clue because the focal plane for blue is in front of the focal plane for green and red (*58*). In principle, a comparison of blue spatial contrast versus red spatial contrast should allow determining where the focal plane is. However, although there is solid evidence that longitudinal chromatic aberrations (LCAs) provide important information for guiding accommodation (*58*), it is less clear for emmetropization. Recent works suggest that modifying the chromatic content can help slow down eye growth in humans (*59*, *60*), but other works suggest that monochromatic light is enough to regulate eye growth (*34*). At the cellular level, several types of cells in the retina have been shown to compare ultraviolet and green signals (*61*, *62*), in particular in natural stimuli (*63*). A detailed simulation of chromatic aberration at various eccentricities remains to be done, but it might be possible to combine contrast cues and chromatic cues to refine even more the estimate of the sign of defocus (*58*). Following our “hybrid” approach, future works could simulate whether chromatic aberrations in the periphery of the retina produce asymmetric PSFs, with a difference that can be detected by these cell types.

### Implication for myopia control strategies

We do not have causal evidence suggesting that our strategy relying on spherical aberrations and LSC is actually the strategy used by the retina to estimate where the focal plane is to modulate eye growth accordingly. An alternative could be that the LSC is just one of the cues used by the retina to detect the sign of defocus and regulate eye growth. However, in both cases, our study gives several predictions that have been verified by previous studies, and it also gives a possible explanation to several observations made in the literature about myopia. Myopia is an excess of eye growth and an important public health concern.

Many studies have aimed at mitigating this health issue by slowing down eye growth (*64*). Among them, it has been proposed to have patients wearing glasses with a mild decrease in contrast in the periphery (*65*), a strategy that can slow down eye growth partially (*66*). If the retina relies on LSC to determine the sign of defocus, as we proposed, a mild decrease in LSC is equivalent to displacing the focal plane in front of the retina. Our proposed strategy predicts that this decrease in LSC should slow down eye growth because it is equivalent to an image focused in front of the retina, which should trigger a reaction to “bring back” the focal plane to focus and thus achieve emmetropization. This is observed in patients: Wearing these glasses slows down the axial growth of the eye (*66*). In addition, optical analyses suggest that other types of lenses that are efficient at slowing down myopia progression in children also decrease the contrast on the retina (*67*–*69*). Our strategy thus provides an explanation for the partial success of these therapeutic strategies: The retina could have its estimate of the focal plane biased to be more in front than it is and would therefore slow down the eye growth to compensate for this.

A major factor driving eye growth is the reduced illumination associated with indoor spaces (*70*) because intensity and spectral composition of ambient light have also been shown to modulate eye growth (*7*, *34*). However, other factors are also associated with an excessive growth of the eye. A factor that seems to increase the probability of being myopic is near sight vision (*71*), although it is always hard to separate from other factors like the level of illumination (*70*). Near sight vision reduces the amount of spherical aberration (*47*), and we have shown that this makes the determination of the sign of defocus more difficult. One possible explanation for the involvement of near sight as a risk factor for myopia might be due to reduced spherical aberrations, and thus the depth of focus will be more difficult to estimate. However, other factors that are correlated with near sight vision, like a decrease in the level of illumination, are also associated with higher risks of myopia and probably also play an important role. Conversely, orthokeratic treatments, which can mitigate strong myopia, increase spherical aberrations (*72*). Overall, although we cannot prove a direct causal link, our strategy provides an explanatory basis for several studies aiming at slowing down eye growth to mitigate myopia.

### Detection of the sign of defocus for accommodation

In principle, our proposed strategy could also be used to determine the sign of defocus for accommodation. However, the accommodative reflex might also rely on several monocular cues (*1*). Some studies have suggested that spherical aberration might be used to find the location of the focal plane during the accommodative reflex (*3*). However, accommodation differs from emmetropization in several ways. First, it acts on a much faster timescale. There will thus be less time to average over images and obtain an accurate estimate of the defocus. Second, accommodation seems to rely more on the visual input at the center of the retina, whereas emmetropization seems to rely more on the periphery. Third, accommodation also relies on visual processing beyond the retina, in subcortical and possibly cortical areas. The role of the retina in the accommodative reflex might thus be to preprocess the input, rather than to perform a complete estimation of the defocus.

### Relationship with form deprivation myopia

One issue with our proposed strategy to estimate the sign of defocus for emmetropization is that it seems at odds with the results obtained with form deprivation myopia (FDM). Several studies have shown that a strong decrease in contrast, usually induced by placing a transducent diffuser in front of the eye [see (*7*) for a review], increases eye growth. Hence, it was hypothesized that increasing the contrast on the retina could inhibit eye growth (*6*, *29*). However, these protocols of form deprivation usually induce a severe loss of contrast and acuity. How FDM depends on the level of attenuation of contrast and spatial frequency content is still unclear. It seems to depend on the exact protocol and on the species (*9*, *73*–*75*). Compared to the studies where a mild decrease in contrast has been shown to slow down eye growth [see figure 10A in (*67*)], FDM protocols induce a much stronger loss of contrast [see figure 2 in (*74*)], which is far outside of the range of loss of contrast that can be induced by a change in focus as simulated in our experiments. It is thus plausible that FDM taps into a very different mechanism than the small loss of contrast that we simulated in our experiments and has thus consequences that are not related. This is further corroborated by several studies suggesting that the mechanisms of FDM and emmetropization are different (*6*).

Using spherical aberrations to determine the sign of defocus has already been proposed by Thibos and colleagues (*46*). However, their conclusions were different from our work: They assumed that a decrease in their “retina contrast” would be equivalent to a focus behind the retina, and would therefore increase eye growth. Their interpretation was based on previous works using FDM, which lead them to assume that lower contrast should increase eye growth. Our results suggest on the contrary that lower contrast on the retina is associated with reduced eye growth, which is corroborated by recent results on myopia control glasses.

A shortcoming of our study is that it was based on investigating how ganglion cells respond to flashed natural images. Although this can approximate how ganglion cells will respond to stimuli right after a saccade (*76*–*78*), more complex types of temporal dynamics are present during natural visual experience. Specifically, between saccades, the visual input will be animated by fixational eye movements, which will strongly reshape the visual input (*79*). Other types of motion might also affect retinal processing. Understanding how the retina responds to animated movies is still an active area of research. Future studies will have to understand whether these temporal dynamics, which are susceptible to tap into more complex mechanisms in retinal processing like adaptation, and will require more complex models, can affect how the retina can extract the sign of defocus.

## MATERIALS AND METHODS

### Mouse eye model

**Fig. 7.**
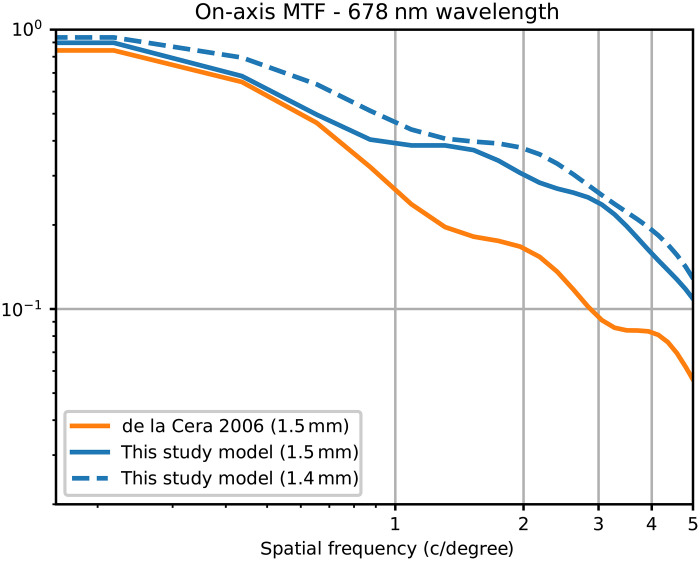
MTF radial profiles of the mouse eye model, simulated on-axis at a wavelength of 678 nm. Comparison with the results of de la Cera and colleagues (*43*) is made for a 1.5-mm pupil diameter, specifically from Fig. 4, line “mouse with defocus (1.5 mm).” Moreover, the profile for a 1.4-mm pupil is shown, which corresponds to the pupil size of interest in the current study.

An optical eye model requires geometrical models for each refractive surface (anterior and posterior cornea and anterior and posterior crystalline lens), relative positioning of each surface (translations and optionally tilts) and the refractive index of each medium. We used data from Schmucker and Schaeffel (*80*), limited to spherical diopters, as a starting point for our optical eye model. This paper provides linear regressions as a function of age (from P22 to P100) for each parameter (averaged over *N* = 20 mice) in the mouse eye. We used the values of the parameters at *t* = 100 days. Next, with a classical ray-tracing approach, we were able to calculate the wavefront error (a function which quantifies how much the optics depart from that of a perfect lens), the PSF (using the Fraunhofer diffraction framework which predicts that the PSF can be obtained from the wavefront error) and the modulation transfer function (MTF; from the Fourier transform of the PSF, it is a function that assesses the ability of the optical system to transfer the contrast from object to image) of the optical eye model. The model parameters, described in the table below (Table 1), were fine-tuned through an optimization process to match the on-axis MTF at very low spatial frequencies, as measured by de la Cera and colleagues (*43*) (Fig. 7). A reasonable match near 0.5 cycles/degree is important because it corresponds to the visual acuity of mice (*81*). Moreover, our model presents a primary spherical aberration of 0.42 μm for a 1.5-mm pupil at a 678-nm wavelength, as quantified by Zernike coefficient $c_{0}^{4}$ , whereas (*43*) measured an average of 0.15 μm. A conic constant was later added as a parameter to the anterior cornea to investigate arbitrary spherical aberrations quantities, which allowed us to verify that similar effects were observable with lower amounts (see fig. S2). Last, we introduced a dispersion formula for the crystalline lens to allow its refractive index to vary with the wavelength. It results in an LCA of 7.9 D between 457 and 633 nm, consistent with the findings of Geng and colleagues (*42*).

**Table 1.** Mouse eye model parameters. The radius is the radius of curvature of the surface. The conic constant is the Zernike coefficient $c_{0}^{4}$ . The distance is the distance from the next surface, starting from the anterior cornea. The retina has no associated distance because it is the last surface in the model.

Component	Radius (mm)	Conic constant	Distance (mm)	Refractive index
Anterior cornea	1.408	−1.38 + 3.15 × $c_{0}^{4}$	0.092	1.402
Posterior cornea	1.372	0	0.278	1.334
Anterior lens	1.150	0	2.004	1.561 + 4.837 × 10^3^/$\lambda_{\text{nm}}$^2^
Posterior lens	−1.134	0	0.942	1.333
Retina	−1.598	0	–	–

The PSFs are calculated using custom software. The simulation parameters include light source eccentricity, pupil size, and defocus. To efficiently account for defocus, the image plane (i.e., the retina) is offset using the properties of the Fourier transform. The defocus ranges from −200 to +200 μm, which corresponds respectively to shifting the retina in front of (behind) of the focal point and therefore to a hyperopic (myopic) defocus. In the mouse, it corresponds to defocus ranging from +40 to −40 D.

### Human eye model

The human eye model is based on the model of Atchison (*82*), which has the specificity of having the ametropia as an input parameter. The conic constant of the anterior cornea was adjusted to match recent population spherical aberration data [mean $c_{0}^{4}$ = 0.18 μm for a 6-mm pupil from (*83*) compared to 0.10 μm in the Atchison model]. Moreover, the shape of the crystalline lens was also modified to correct the higher than observed peripheral astigmatism (*84*). As for the mouse eye model, the light source eccentricity, the pupil diameter and thee defocus can be varied. The defocus ranges from −200 μm (+0.68 D) to +200 μm (−0.68 D). To simulate the accommodation process, the central curvature of the anterior lens was updated according to the stimulus proximity so that paraxial rays coming from an object point at near focus on the fovea.

### Polychromatic PSFs

The eye models described above predict monochromatic PSFs at a given wavelength. As we wanted to simulate retinal images as seen by an eye watching at a natural scene, we computed more ecological PSFs using the sensitivity curves of the different types of cones, as follows. For the mouse, we considered only the M cone (peak intensity at 508 nm) and simulated PSFs for wavelengths ranging from 408 to 608 nm, every 10 nm. For the emmetropic human, we considered the M and the L cones (peak intensity at 537 and 567 μm, respectively) and simulated PSFs for wavelengths ranging from 450 to 650 nm, every 10 nm. To obtain the polychromatic PSFs, we then computed a weighted sum of the monochromatic PSFs, where the weight is the cones absorption value at the given wavelength. Note that, to design our stimuli for mice, we did not include rods because their spectral sensitivity is very similar to that of M cones in mice. However, as it was recently shown that the rod pathway might be important for eye growth control in mice (*85*, *86*), exploring rod’s involvement in more detail could be a future improvement of our model.

### Electrophysiological recordings

All experiments were performed in accordance with institutional animal care standards of Sorbonne Université (license number C-75-12-02). We used six C57BL/6J mouse retinas. After the euthanasia of the animal, the eye was enucleated and transferred into oxygenated Ringer medium (125 M NaCl, 2.5 M KCl, 1 M MgCl_2_·6H_2_O, 1 M CaCl_2_, 0.43 M l-glutamine, 1.25 M NaH_2_PO_4_, 20 M glucose, and 26 M NaHCO_3_) or Ames medium. Dissection was performed under dim light conditions. A piece of retina was mounted onto a dialysis membrane and then lowered with the ganglion cells toward the recording system. Retinas were kept at 36° during the whole experiment.

Electrophysiological recordings of the ganglion cell’s spiking activity were subsequently performed with a MEA (252 electrodes, 30 μm in distance between the electrodes, and 8 μm in diameter) as described in (*45*). In brief, the raw signal was acquired through MC_Rack Multi-Channel Systems software V 4.6.2 with a sampling rate of 20 kHz and high-pass filtered at 100 Hz. Spikes were isolated with SPYKING CIRCUS V1.0.9. Subsequent spikes analysis was performed with custom-made Python codes. We kept cells with less than 0.6% refractory period violation (2-ms refractory period). Cells that did not show a clear response to the white-noise stimuli (see below) were discarded because it prevents estimation of the receptive field and therefore the calculation of the LSC. In addition, we also discarded cells with a very low response to the chirp stimuli (see below) as it is needed for cell typing.

### Visual stimulations

A white mounted light-emitting diode (MCWHLP1, Thorlabs Inc.) was used as a light source, and the stimuli were displayed using a digital mirror device (DMD; DLP9500, Texas Instruments) and focused on the photoreceptors using standard optics and an inverted microscope (Nikon). The light level corresponded to photopic vision: 3.90 × 10^3^ and 1.48 × 10^6^ isomerizations/(photoreceptor·s) for S cones and M cones, respectively.

#### Checkerboard stimuli

We displayed a random binary checkerboard during 40 min to 1 hour at 30 Hz to estimate the position and the size of the receptive fields of ganglion cells, as described in (*45*). The check size was 42 μm, and checkerboards had a size of 60 x 60 checks. Receptive fields were estimated using the classical “spike-triggered average” (STA) method. We fitted a 2D Gaussian function on the resulting spatial STA and defined the receptive field as the ellipse corresponding to the 2σ contour of the fit.

#### Natural image stimuli

We used the Open Access van Hateren Natural Image Dataset (*87*), which consists of 4212 monochromatic and calibrated images taken in various natural environments. Images were preprocessed as explained in (*45*).

The dataset of 3190 images remaining after the preprocessing step was used to stimulate the retina. We selected 30 images that were presented 30 times to create the test dataset for the CNN. The other images were only flashed once. Around 10% of them (250 images) were allocated to the validation dataset, whereas the rest of the images composed the training dataset.

#### Defocused images stimuli

To generate the defocused images, we selected four images in the above dataset and convolved them with the polychromatic PSFs obtained with the mouse eye model described above. The size of the pixels of the images corresponds to the size of the pixels of the DMD (3.5 μm). Therefore, we first had to upscale the images to match the pixel size of the PSFs (1 μm). The upscaled image was convolved with the PSF using the fftconvolve function from the Scipy Python package. Then, the convolved image was downscaled to match the DMD pixel size (3.5 μm). Upscaling and downscaling were performed with cubic spline interpolation. Because of the position of the peak of the PSF that can change from one PSF to the other, some convolved images were shifted with respect to the original image. We realigned the convolved images on the original image to ensure that we recorded responses to a change of optical transformation and not to a shift of the image. Realignment was done by maximizing the correlation between the “contours Fourier image” of the convolved image and the original. Briefly, Fourier transform was performed, low frequencies were filtered out, and the inverse Fourier transform was applied, a similar process which is typically used to align calcium imaging data recorded with a two-photon microscope. In some cases, small man-made adjustments were needed to ensure accurate realignment. Each defocused image was presented 25 times to the retina, randomly shuffled with the sharp natural images.

Images were displayed during 300 ms, followed by a gray frame presented during 300 ms. To obtain RGC’s response to the images, we counted the spikes occurring between 50 and 350 ms after the start of the image presentation. To measure the proportion of images leading to a strictly negative or strictly positive change of firing rate, as in Fig. 2F, for each cell, each image and each optical condition, we compute the difference of firing rate between defocus 200 and defocus −200 μm. Then, for each optical condition, we count the number of cells and images for which the difference is smaller than zero (“strictly negative”) or larger than zero (“strictly positive”).

### Cell typing

The cell typing procedure is described in detail in (*45*, *88*). In brief, in addition to the white-noise stimuli and the natural images, we displayed an additional full-field “chirp” stimulus (Fig. 2A), similar to the one used by Baden and colleagues for their RGC classification (*44*). It is composed of ON and OFF steps of varying amplitude and frequencies with values ranging from 0 to 1. The chirp was presented 20 times during 32 s at a frequency of 50 Hz.

For typing the cells, we clustered the RGCs based on their response to the chirp stimuli and their STAs (*45*, *88*). It resulted in RGC groups that we assigned to 1 of the 32 types described in (*44*). To do so, we transformed our peristimulus histograms in calcium events by convolving them with a decaying exponential (*45*, *88*). Cell types that present strong responses to the modulating frequencies and amplitude were assigned correctly, whereas other types, which mostly respond to ON/OFF steps, were assigned in a second round of correlation match. Besides the correlation of the chirp traces, we confirmed the correct assigning of groups by checking that the ellipses of each type form a proper mosaic, that the temporal STAs look uniform, and the similarity of the spike waveforms. Last, we correlated the chirp’s responses across experiments to ensure homogeneous groups.

### Ganglion cells modeling criteria

We modeled cells from retinas in which we found OFF slow and ON-OFF local cells (three of six retinas) and selected the cells following two criteria: (i) having a proper STA and (ii) having a stable response to the 30 sharp images that were presented 30 times randomly across the experiment. Stability was assessed using the criterion of Cadena and colleagues (*89*). We computed the ratio between the explainable and the total variance and discarded all the cells with a ratio smaller than 0.22. In total, we were able to model 82 cells from three experiments. These two criteria explain the discrepancy between the total number of OFF slow, ON-OFF local, and ON alpha cells (Fig. 2) and the number of cells that were modeled in the CNN model (Fig. 3). Because the defocused and not defocused images were shuffled randomly during the experiments, we are sure that the change of activity that we measure in response to defocus comes from defocus and not from unstable cell’s activity.

### CNN model

The architecture of the CNN model is described in detail in (*45*). Briefly, it is a two-layer network. The first layer is a convolutional layer made of four 2D convolutional kernels. The filters are learned from the data independently for each experiment and common to all cells. The second layer is made of one filter for each cell, followed by a nonlinearity.

The model was fitted on the data by minimizing a loss function on the training set, as explained in (*45*). The training set is made of 2910 not defocused natural images and the corresponding RGC responses (number of spikes from 50 to 350 ms after the start of the presentation of the image).

To evaluate the performance of the model, we used a testing set composed of 30 not defocused natural images that were presented 30 times and the associated RGC responses. Using repeated images allows to discriminate between the error due to the limitations in the model and the error due to the intrinsic noise in the RGC responses. We then calculated the noise-corrected correlation as introduced in (*90*) and detailed in (*45*).

### Classical LN model and SC model for mouse data

For this analysis, some additional cells were discarded because their STA was inaccurately fitted, and to measure the LSC, receptive field estimation needs to be accurate. We consider the local image statistics, which are MI and the LSC in the RGC’s receptive fields. The receptive field is the 2σ contour of the 2D Gaussian fit *G*(*x*) (see the Materials and Methods section “Visual stimulations”). Therefore, we consider only the pixels within the ellipse and transform the light level of each pixel in a Weber contrast: *C* = *L* − *L*_mean_/*L*_mean_, where *L*_mean_ is the mean light level over the entire image (*40*).

We used the classical LN model and the SC model developed by Liu and colleagues (*40*). We did exactly as they describe in their methods; therefore, we give only a brief description of the models here.

Both models start with spatial filters applied to the stimulus, *F*_LN_ and *F*_SC_, for the classical LN and the SC model, respectively. For each image, *F*_LN_ is the MI in the receptive field, *I*_mean_

$$I_{\text{mean}}=\frac{1}{N}\sum_{i=1}^{N}G\left(x_{i}\right).C\left(x_{i}\right)$$

where the sum is taken over the *N* pixels positions *x*_i_ located within the receptive field [2σ contour of the 2D Gaussian fit *G*(*x*)], *G*(*x*_i_) is the weight of pixel *i*, and *C*(*x*_i_) is the Weber contrast of pixel *i*. *F*_SC_ receives an additional input, the LSC (LSC), which is the SD of the weighted pixels intensity in the receptive field

$$\text{LSC}=\sqrt{\frac{1}{N-1}\sum_{i=1}^{N}{\left[G\left(x_{i}\right).C\left(x_{i}\right)-I_{\text{mean}}\right]}^{2}}$$

The LSC is added to *I*_mean_ with a weight *w* to obtain *F*_SC_

$$F_{\text{SC}}=I_{\text{mean}}+w\cdot \mathrm{LSC}$$

The firing rate was then predicted from *F*_LN_ and *F*_SC_ by passing them through a nonlinear parameterized “softplus” function

$$r\left(F_{X}\right)=a\cdot \text{ln}\left[1+e^{b\;\left(F_{X}+c\right)}\right]$$

where *F*_X_ stands for *F*_LN_ or *F*_SC_. Both models were trained and tested with the ganglion cell’s response to the not defocused images only. For each model, the responses to a training set of 3160 not defocused natural images were used to fit *r*(*F*_X_). The parameters *a*, *b*, and *c* were optimized with the curvefit function from Scipy with 50 iterations to find the initial values leading to the smallest residual error. The fitted functions were then used to predict the cell’s responses to the test set (30 not defocused natural images repeated 30 times). To quantify the model performance, we computed the coefficient of correlation *R* between the predicted response and the measured spike count for each image in the test set and reported the explained variance *R*^2^.

### MI and LSC in human retinal images

To obtain the LSC in natural images transformed with the eye optics of a human eye, we defined a generic receptive field as a 2D Gaussian. The size of the receptive fields was chosen based on measurements in human retina (*91*). The receptive field of human parasol cells ranges roughly from 100 to 200 μm, with a median at 144 μm for ON cells and 123 μm for OFF cells. Human midget cells have a receptive field diameter ranging from roughly 30 to 100 μm, and the median for ON cells is 64 and 46 μm for OFF cells. We used 120 μm as the standard size for the 2σ contour of the Gaussian fit. We then get the pixels comprised within that contour as an array of coordinates and compute the LSC with the above formula. We verified that, for the large majority of images, the method is robust to a variation of the size of the receptive field, within the range of the measured diameters (30 to 200 μm).
