# Supplementary material for: Nonlinear spatial integration allows the retina to detect the sign of defocus in natural scenes
Source: Sci Adv. 2025 Aug 8;11(32):eadq6320. doi: 10.1126/sciadv.adq6320 (PMC12333688; doi:10.1126/sciadv.adq6320)
Supplement: Supplementary file 1 — Text S1 Figs. S1 to S6 [file sciadv.adq6320_sm.pdf]

Science Advances
AAAS

Supplementary Materials for

Nonlinear spatial integration allows the retina to detect the sign of defocus in natural scenes

Sarah Goethals *et al.*

Corresponding author: Sarah Goethals, goethas@essilor.fr; Olivier Marre, olivier.marre@inserm.fr

Sci. Adv. **11**, eadq6320 (2025)
DOI: 10.1126/sciadv.adq6320

This PDF file includes:

Text S1
Figs. S1 to S6

Text S1 (with supplemental figure S5)

We propose a strategy for the retina to find the focal point. The retina could average the local spatial contrast (LSC) over time and space, and depending on how this averaged value compares to a reference value LSC_0, which is the average LSC at the focal point, the retina could determine where the best focus is. To test if this is a viable strategy, we calculated the mean LSC over N random samples and found that it changes monotonically with defocus in the close vicinity of the focal point (defocus = 0 µm), both for central and peripheral eye optics (fig. S5 A). For a large region around a defocus of 0, the curve is monotonic, which shows that measuring the average LSC can allow estimating the depth of focus unambiguously.

However, since the estimation of the LSC might be prone to error, this strategy might lead to error in the estimation of the defocus. This could end up with an error on the location of the focal point. We thus estimated the error made when using the LSC to determine the error in the estimation of the focal point. For each defocus value, we obtained the distribution of the mean LSC and computed the probability, for each defocus, that the estimate of the mean LSC equals the reference value LSC_0, i.e. the value of LSC at the focal point for a "perfect" estimator (fig. S5 B). From this distribution we could estimate the average error in the localization of the focal plane made when using this strategy (fig. S5 C). This error depends on the number of samples used for averaging, but, assuming this number is large enough (N above 2000), this gives an error below 0.08 D, which seems acceptable to regulate eye growth.

Note that, in the case where the activity of a cell depends both on the mean intensity (MI) and the LSC, it remains possible to decode the LSC value from a population of neurons. There are cells that are driven more by MI than LSC: we have shown that this is the case of ON alpha cells, based on previous work (fig. S1, ref. (46)). Goldin and colleagues have also shown that some RGCs respond only to a change in luminance in their receptive field, and not to a change of contrast (fig. 4D of (46)). Karamanlis and Gollisch made a similar observation (59). From their activity, it is possible to disambiguate the response of cells that respond both to LSC and MI, and obtain the value of LSC. However, to fully uncover the mechanisms that allow estimating the sign of defocus, we would need to know precisely which cell types are causally involved in this estimation, which is beyond the scope of this study.

Fig. S1.

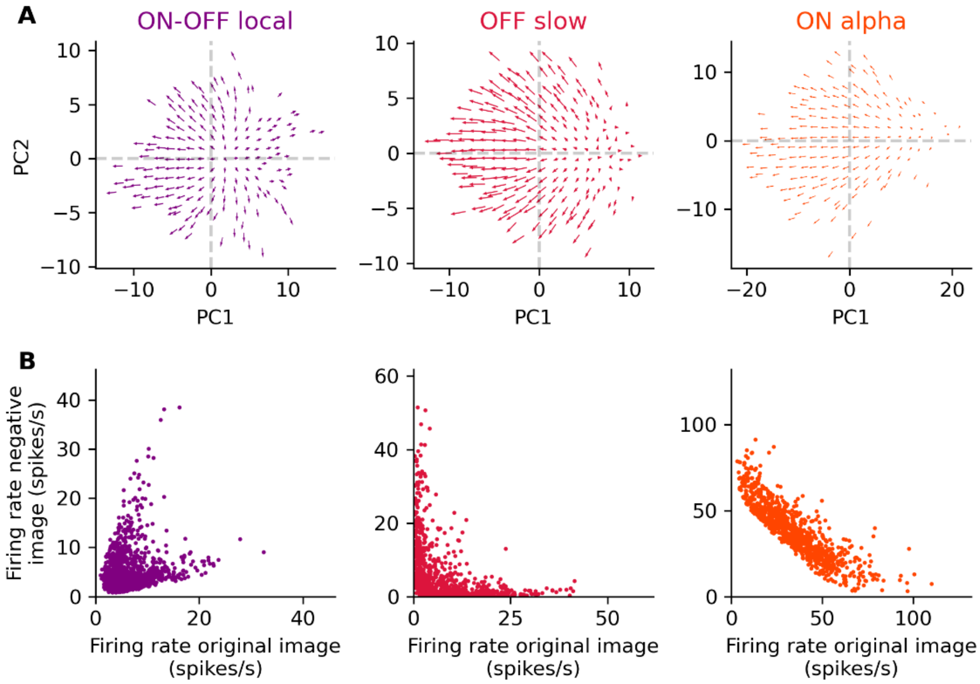

Defocus detectors behave like contrast encoders (46) **or encode a mix of mean intensity and LSC.** Left: ON-OFF local, middle: OFF slow, right: ON alpha. **A,** Vector fields that illustrate the best subtle image modifications that would increase the cell's firing rate response. To build these plots, we use the CNN model to predict, for each image, the Local Spike-Triggered Average (LSTA, ref. (46)), which describes how to modify the image to increase the firing rate of a cell. These LSTAs are high-dimensional, but a Principal Component Analysis (PCA) reduces them to two main axes (PC1 and PC2) that capture most of the variability of each cell. In these 2D spaces, each dot represents an input image, and the corresponding arrow shows the direction (in PCA space) that would increase the firing rate (see Fig. 4 of ref. (46) and Methods of ref. (46)). ON-OFF local cells show outward-pointing vector fields centered near the origin (typically a uniform gray), characteristic of contrast detectors (46): firing increases with both brightness and darkness away from the mean gray level. ON alpha cells, on the contrary, show aligned vectors pointing along a single axis, indicating that firing increases with a consistent change in overall luminance (e.g., becoming brighter). OFF slow cells show an intermediate pattern, combining features of contrast (LSC) and luminance sensitivity (mean luminance). **B**, firing rate at zero defocus in response to the negative images vs. firing rate at zero defocus in response to the original images, for simulated central eye optics (0°). Each dot represents an image.

Fig. S2

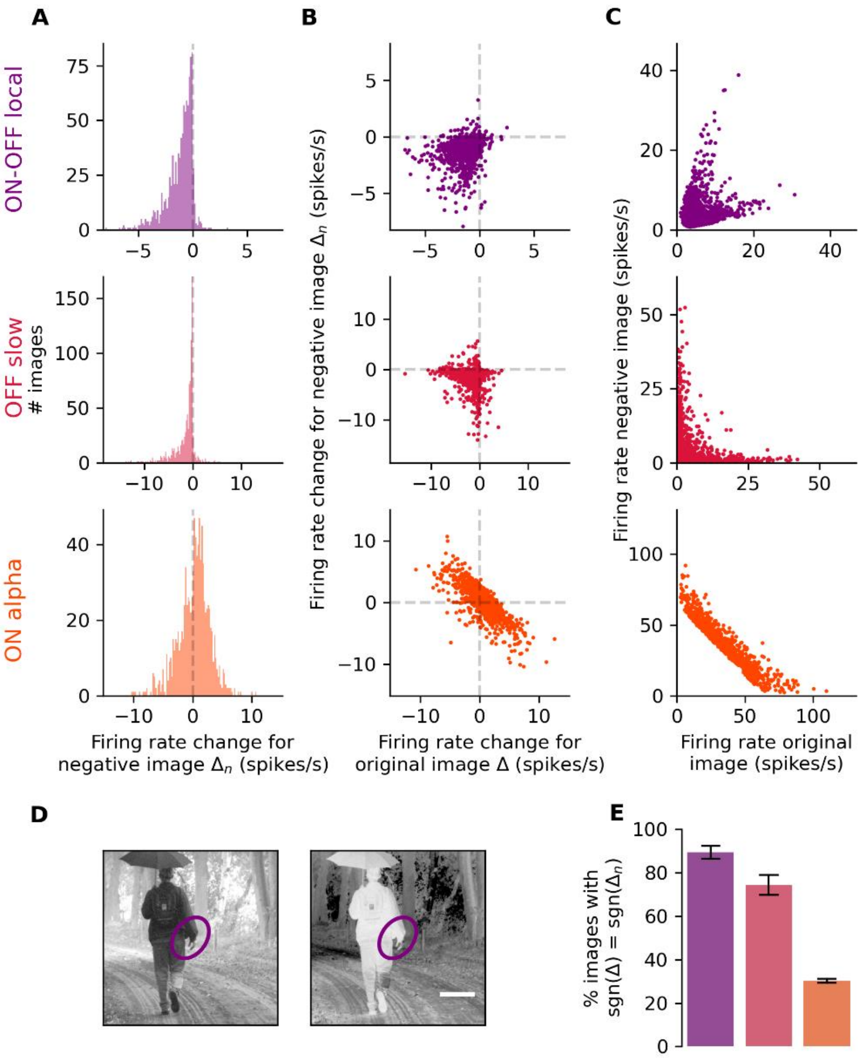

A simple contrast model and negative images confirm that defocus detectors encode contrast, for simulated peripheral eye optics (20°). A, distribution over 1000 images of the change of firing rate between 200 µm and –200 µm in response to the negative defocused images. Top, example ON-OFF local cell. Middle, example OFF slow cell. Bottom, example ON alpha cell. **B**, change of firing rate in response to the negative images vs. change of firing rate in response to the original images for the same cells as in panel **A**. Each dot represents an image. **C**, same as **B** but for the firing rate on the retina (defocus = 0D). **D**, an example natural image (top) and its bright-dark inversed image (bottom). The ellipse represents the receptive field of an ON-OFF local example cell (same cell as in panels A, B and C). **E**, average (over cells) of the proportion of images leading to a firing rate change Δ_n (between defocus of +200 µm and defocus of –200 µm), that as the same sign as the firing rate change Δ for the original image. Left, ON-OFF local (N = 3); middle, OFF slow (N = 4); right, ON alpha (N = 15). Data is represented as mean ± SEM.

Fig. S3.

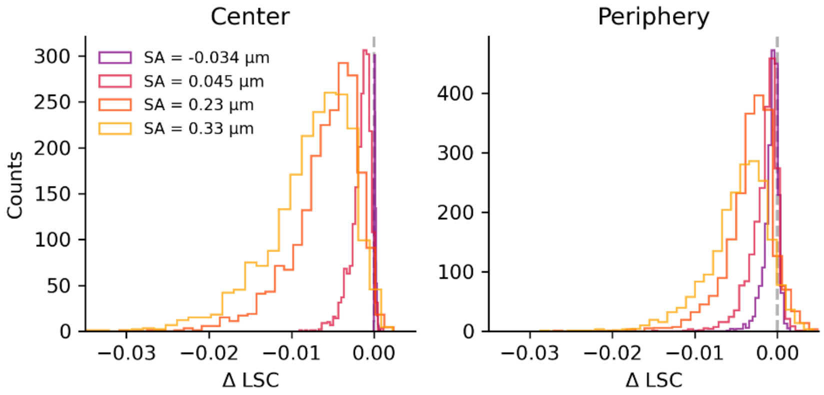

Effect of variations of the amount of spherical aberrations in the mouse eye model on the local spatial contrast (LSC). Distribution over N = 511 cells x 4 images of the difference in LSC between a defocus of 200 µm and a defocus of -100 µm. Left, for simulated central eye optics (0°). Right, for simulated peripheral eye optics (20°).

Fig. S4

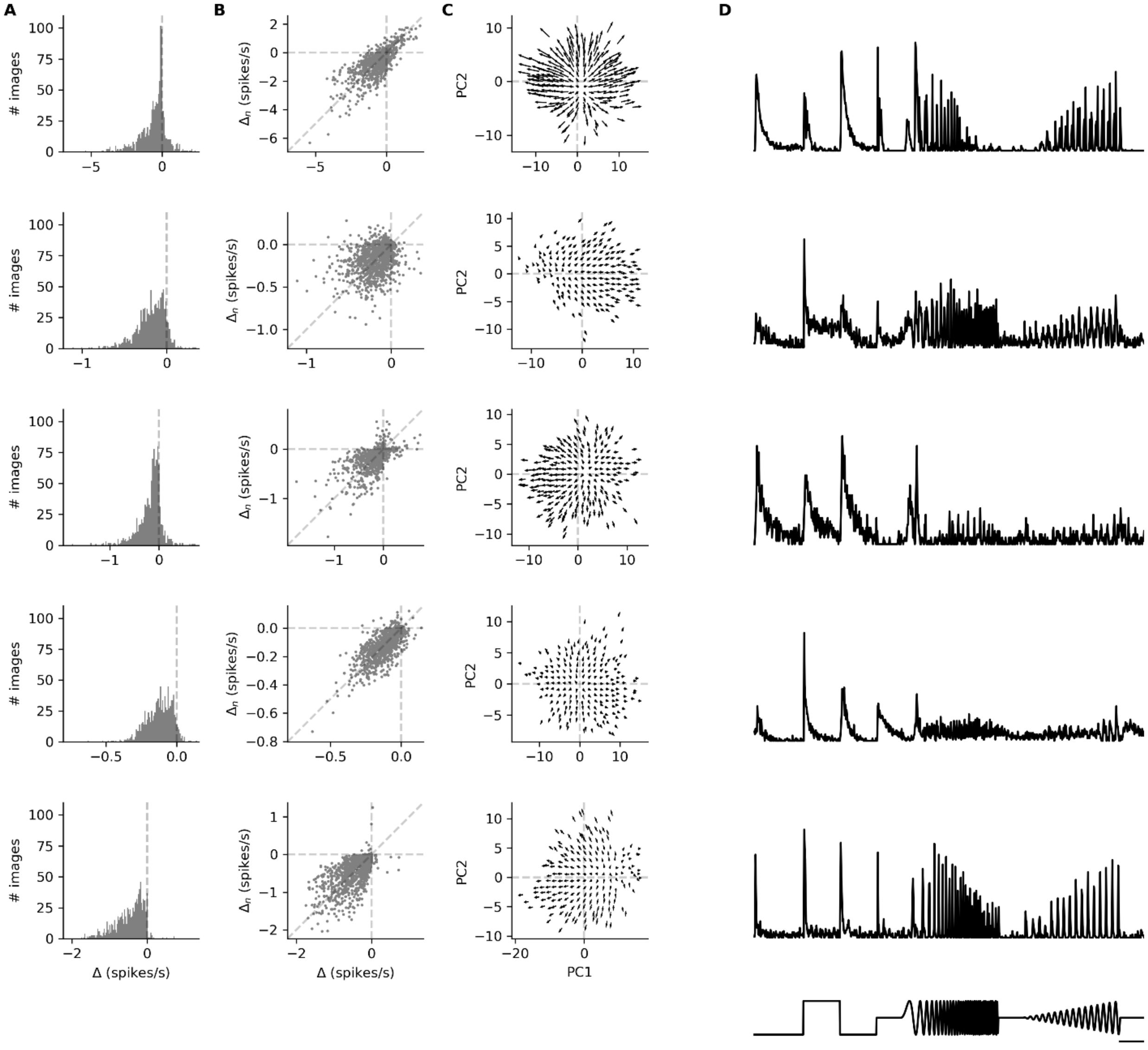

Defocus detectors can belong to other types. In addition to the OFF slow and ON-OFF local types in which all the cells are defocus detectors, we found defocus detector cells in other types. Each line corresponds to a different defocus detector cell. **A**, distribution over 1000 images of the difference in firing rate between positive (+200 µm) and negative (-200 µm) defocus. **B**, change of firing rate for the negative image vs. change of firing rate for the original image, for 1000 images. Each dot represents an image. **C**, vector fields (see Fig. 4 of ref. (46) and Methods of ref. (46)). The first, the third and the fourth cells have diverging vector fields centered on the origin, indicative of LSC encoding (46). The second and fifth cells have diverging vector fields centered further away from the origin, indicating a mixture of mean intensity and LSC encoding (46). **D**, normalized response of the defocus detector cells to the bright-dark chirp (bottom trace). Scale bar: 2 seconds.

Fig. S5

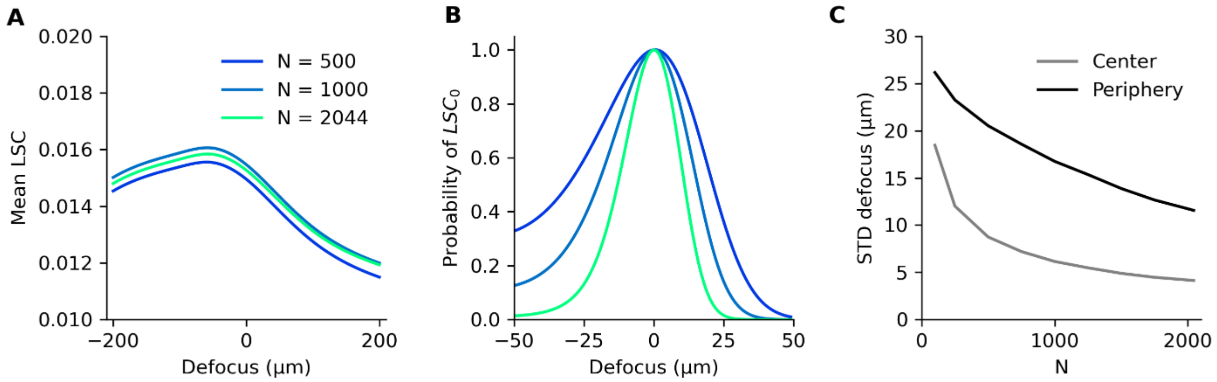

A strategy to estimate the position of the best focus. A, mean local spatial contrast (LSC) as a function of defocus for different sample sizes N and for simulated peripheral eye optics (20°). **B**, for a given defocus, the probability for the averaged mean LSC to equal LSC_0, the averaged mean LSC at the focal point (0 µm). This probability is shown for different sample sizes N for simulated peripheral eye optics (20°). **C**, error on estimating the position of the focus with the averaged mean LSC, for different sample sizes and for simulated central (grey) and peripheral (black) eye optics.

Fig. S6.

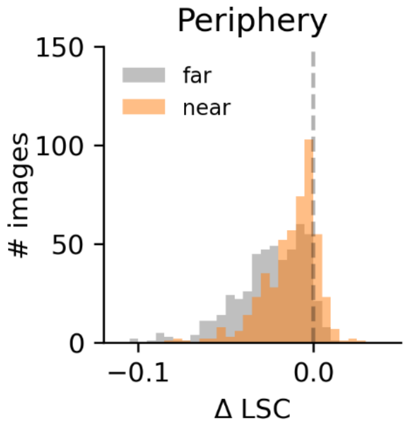

Near vision makes local spatial contrast more ambiguous to determine the sign of defocus with peripheral eye optics . Distribution of the difference in LSC between 200 µm and -200 µm over N = 500 images, for peripheral eye optics (20°=) and for a model with spherical aberrations and in far vision (grey, same as the grey histogram of fig. 6D right) and a model with spherical aberrations in near vision (orange, focus proximity = 2.5 D, SA = 0.030 µm).
